# The revisional burden of colostomy: a temporal analysis of risk, patterns, and survival

**DOI:** 10.1007/s10151-026-03328-2

**Published:** 2026-05-05

**Authors:** Medeni Sermet, Ozgur Ekinci, Orhan Alimoglu

**Affiliations:** Department of General Surgery, Goztepe Prof. Dr. Suleyman Yalcin City Hospital, Istanbul Medeniyet University, Istanbul, Turkey

**Keywords:** Colostomy, Stoma revision, Stoma complications, Parastomal hernia, Stoma necrosis

## Abstract

**Objective:**

This study analyzed patients who underwent colostomy revision to identify risk factors and complications, aiming to provide evidence-based recommendations for the guidelines.

**Materials and methods:**

Of 339 colostomy patients treated between 2016 and 2023, 58 who underwent colostomy revision were designated as the study group and compared with 281 non-revision patients in the control group.

**Results:**

Of the 339 patients, 58 (17.1%) underwent 78 revision procedures (mean: 1.34 revisions per patient). Early revisions (within 30 days) comprised 60.3% of all procedures (*n* = 47), primarily due to necrosis (38.3% of early revisions) and retraction (29.8% of early revisions). Late revisions (after 30 days) accounted for 39.7% (*n* = 31), mainly for parastomal hernia (25.8% of late revisions) and stenosis (19.4% of late revisions). Twenty patients (34.5%) required multiple revisions; the revision group had a higher mean age (66.1 ± 11.8 vs. 61.8 ± 12.1 years, *p* = 0.021), more females (53.4% vs. 38.0%, *p* = 0.045), and more emergency surgeries (58.6% vs. 40.5%, *p* = 0.003). Transverse colostomies had a higher revision rate (24.7%) than sigmoid colostomies (13.8%), *p* = 0.045.

**Conclusion:**

Colostomy revision is associated with significant morbidity and mortality rates. Advanced age, female sex, emergency surgery, and transverse colostomy were identified as independent risk factors. Early complications (necrosis and retraction) differ from late complications (hernias and stenosis). Many patients require multiple revisions, highlighting the need for tailored surgical strategies and updated guidelines to minimize the number of revisions and improve the outcomes.

## Introduction

Colostomy is a frequently employed diversion method in surgical practice and an indispensable component of colorectal surgery. This procedure, which can be performed under both emergency and elective conditions for various indications, significantly impacts patients' quality of life [1]. In the literature, colostomy creation rates are reported in 10–30% of all colorectal surgeries [2, 3]. However, colostomy complications represent a significant problem that adversely affects patients' physical and psychological well-being [4].

Stoma complications can be classified as early or late. Early complications include necrosis, retraction, bleeding, infection, and skin problems, whereas late complications include parastomal hernia, stenosis, prolapse, fistula, and metabolic complications [3, 4]. The incidence of these complications ranges from 20 to 70%, and they can seriously impair the patient’s quality of life, lead to additional treatment costs, and result in prolonged hospital stays [5, 6].

Although some complications can be managed with conservative methods or stoma therapy, many require surgical interventions. Revision procedures can range from local corrections to complete stoma reconstruction [7]. Colostomy revision is generally considered more complex than the initial surgery. Abdominal adhesions, poor general patient condition, and local tissue damage are factors that increase the difficulty of revision surgery [8].

While numerous studies have examined the incidence and risk factors of colostomy complications, comprehensive analyses focusing specifically on cases requiring revision and containing detailed demographic, clinical, and surgical profiles are limited [9, 10]. Although current surgical guidelines provide recommendations for stoma creation, standardized protocols for revision indications, timing, and techniques may be insufficient[11, 12].

The primary aim of this retrospective study was to establish a comprehensive profile of patients undergoing colostomy revision, detail the reasons and types of revision, and elucidate the postoperative outcomes and complication patterns. Considering these findings, this study aimed to develop strategies for identifying high-risk patients and preventing complications, and to contribute to current surgical guidelines regarding revision management.

## Materials and methods

### Study design, ethical approval, and patient selection

**Fig. 1:**
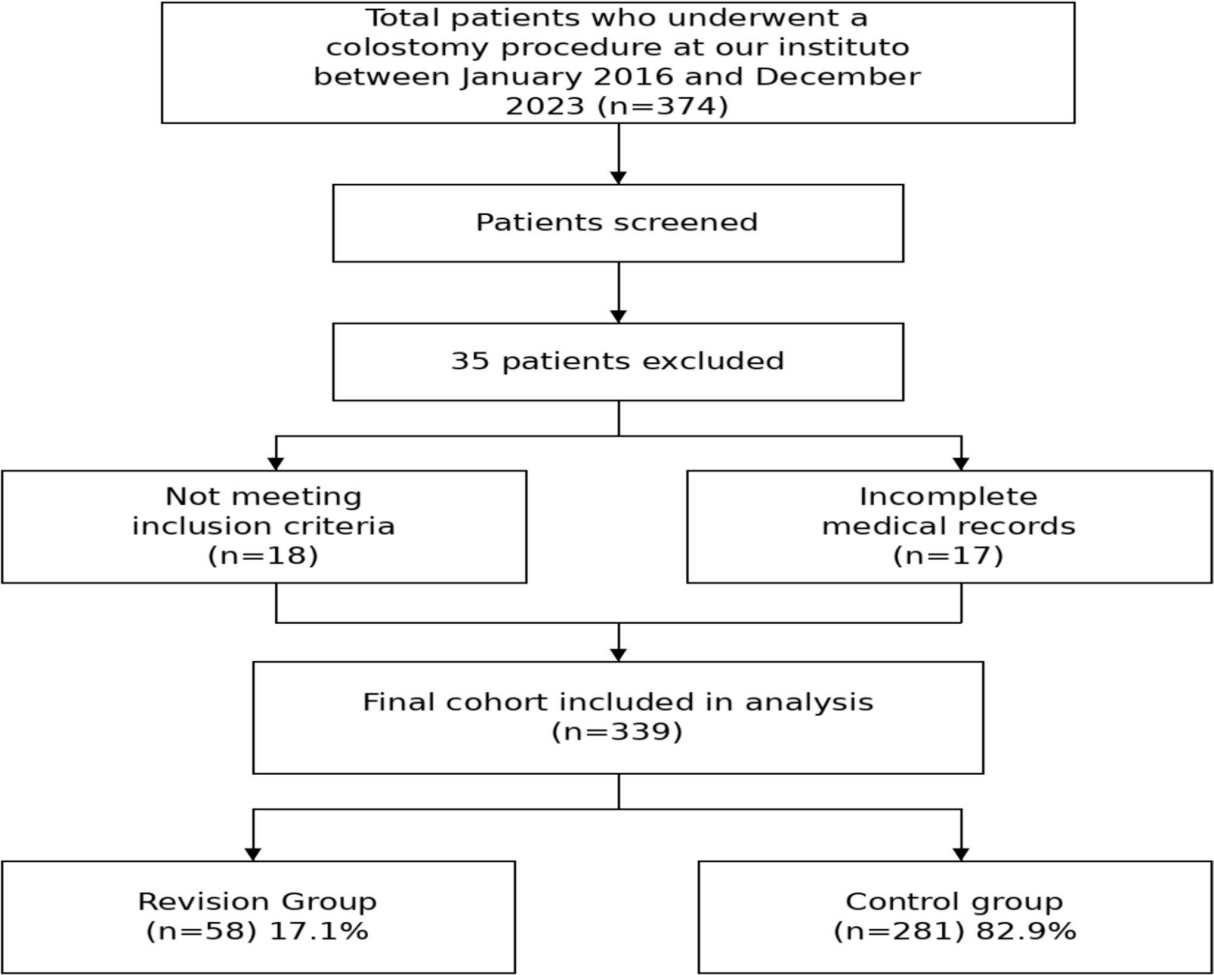
Patient flowchart

This retrospective cohort study followed the STROBE guidelines and received approval from the Ethics Committee of ******University (protocol no. 2025/****). The study included 374 patients who underwent colostomy surgery at the ******* Hospital between January 2016 and December 2023. After excluding 35 patients based on the inclusion criteria (age > 18 years, complete medical records), 339 patients comprised the final cohort (Fig. 1). The revision group comprised 58 patients who underwent at least one colostomy revision, categorized as follows: (1) local revision peristomal interventions without laparotomy; (2) full revision complete stoma reconstruction via laparotomy; (3) laparoscopic correction. The control group comprised 281 patients without revisions. Patients who underwent direct stoma reversal were excluded from the revision group.

### Data collection methods

Data were systematically extracted from electronic medical records by two independent reviewers. The removal process covered a comprehensive set of variables, including patient demographic information, such as age, sex, and comorbidities, and detailed surgical information covering indication, urgency, stoma segment and type, and duration of surgery. Preoperative albumin and hemoglobin levels and perioperative parameters, such as admission to the intensive care unit during the index surgery (first colostomy surgery), were meticulously recorded. Long-term outcomes, such as the types and schedules of complications during data collection, revision procedures (along with specific indications, types, and timings), hospital stay after index surgery, and mortality, were also noted. In addition, long-term outcomes, such as the types and schedules of complications, specific indications, types and schedules, revision procedures, hospital stay, and mortality, were noted during data collection. To maintain data quality and consistency, any discrepancies identified between the two reviewers were resolved by consensus with a senior surgeon. To ensure data quality and consistency, any discrepancies were identified.

### Statistical analysis

Statistical analyses were performed using IBM SPSS Statistics for Windows, version 25.0 (IBM Corp., Armonk, NY, USA). Descriptive statistics are expressed as mean, standard deviation, median, and range for continuous variables and as number and percentage for categorical variables. The normal distribution of continuous variables was assessed using the Shapiro-Wilk test. The chi-square or Fisher's exact test was used to compare categorical variables. To compare continuous variables between the two groups, an independent samples *t*-test was used if parametric conditions were met; otherwise, the Mann-Whitney U test was employed. Multivariable logistic regression analysis was performed to identify the risk factors for revision using the backward elimination method with entry criterion *p* < 0.10 and removal criterion *p* > 0.15. Model fit was assessed using the Hosmer–Lemeshow goodness-of-fit test, and discriminative ability was evaluated using the area under the receiver-operating characteristic curve (AUC). The significance level was set at *p* < 0.05.

## Results

### General patient population and colostomy indications

The indications for colostomy are summarized in Table 1. Malignant causes comprised 69.6% (*n* = 236), predominantly rectal cancer (22.4%, *n* = 76), colon cancer (21.5%, *n* = 73), and gynecological cancer (13.5%, *n* = 46). Other malignancies accounted for 12.1% (*n* = 41). Benign pathologies represented 30.4% (*n* = 103), with diverticulitis (8.3%, *n* = 28), trauma (7.4%, *n* = 25), inflammatory bowel disease (5.6%, *n* = 19), anastomotic leak (4.7%, *n* = 16), and mesenteric ischemia (3.0%, *n* = 10) being most common.

**Table 1:** Distribution of colostomy indications

Indication category	Specific indication	Patient number (*n* = 339)	Percentage (%)
Malignant causes	Total	236	69.6
	Rectal cancer	76	22.4
	Colon cancer	73	21.5
	Gynecological cancers	46	13.5
	Other malignancies	41	12.1
Benign causes	Total	103	30.4
	Diverticulitis	28	8.3
	Trauma	25	7.4
	Inflammatory bowel disease	19	5.6
	Anastomotic leak	16	4.7
	Mesenteric ischemia	10	2.9
	Other benign causes	5	1.5

*n*, number; % percentage. Statistical method: descriptive statistics

### Analysis of demographic and clinical characteristics

The demographic and clinical characteristics are shown in Table 2. The revision group was significantly older (66.1 ± 11.8 vs. 61.8 ± 12.1 years; *p* = 0.021) and had a higher proportion of females (53.4% vs. 37.9%; *p* = 0.045). The median follow-up duration was 28 (IQR: 24–52) months for the revision group and 26 (IQR: 24–58) months for the control group. Emergency surgery was significantly more frequent in the revision group (58.6% vs. 39.3%; *p* = 0.003). Preoperative laboratory values revealed significantly lower albumin (3.2 ± 0.5 vs. 3.5 ± 0.6 g/dl; *p* = 0.028) and hemoglobin (11.5 ± 1.8 vs. 12.2 ± 2.1 g/dl; *p* = 0.035) levels in the revision group. Neoadjuvant chemoradiotherapy rates were 22.4% (*n* = 13) in the revision group and 16.3% (*n* = 48) in the control group (*p* = 0.242), while adjuvant therapy rates were 46.6% (*n* = 27) and 41.2% (*n* = 121), respectively (*p* = 0.432), showing no significant differences.

**Table 2:** Comparative analysis of demographic and clinical characteristics

Variable	Revision group (*n* = 58)	Control group (*n* = 281)	*p*-value
Age (years, mean ± SD)	66.1 ± 11.8	61.8 ± 12.1	0.021
Gender, *n* (%)			0.045
Female	31 (53.4)	107 (38.0)	
Male	27 (46.6)	174 (61.9)	
Emergency surgery, *n* (%)	34 (58.6)	114 (40.5)	0.003
Preoperative albumin (g/dl)	3.2 ± 0.5	3.5 ± 0.4	0.028
Preoperative hemoglobin (g/dl)	11.5 ± 1.8	12.1 ± 1.5	0.035

*SD*, standard deviation; *n*, number. Statistical methods: Student's *t*-test for continuous variables, chi-square test for categorical variables

### Technical characteristics of stoma construction and associated revision

The technical features of stoma construction and their relationship with revision are presented in Table 3. Regarding the stoma segment, transverse colostomies had a significantly higher revision rate (24.7%, *n* = 26 of 108) than sigmoid colostomies (13.8%, *n* = 32 of 231; *p* = 0.045). When stoma type was evaluated, loop colostomies demonstrated a revision rate of 17.6% (*n* = 46 of 261), while end colostomies had a revision rate of 15.4% (*n* = 12 of 78), with a statistically significant difference between the groups (*p* = 0.032).

**Table 3:** Stoma technical features and revision relationship

Technical factor	Total patients (*n* = 339)	Revision patients (*n* = 58)	*p*-value
Stoma segment			0.045
Sigmoid	231	32 (13.8%)	
Transverse	108	26 (24.7%)	
Stoma type			0.032
Loop	261	46 (17.6%)	
End	78	12 (15.4%)	

*n*, number. Statistical method: chi-square test

### Revision complications and timing

The timing of the revisions is presented in Table 4. Early revisions (within 30 days) comprised 60.3% (*n* = 47), whereas late revisions (after 30 days) accounted for 39.7% (*n* = 31; *p* = 0.012). Some patients underwent multiple procedures, resulting in 78 procedures in 58 patients, with a mean of 1.34 revisions per patient.

**Table 4:** Revision timing

Characteristic	Patients (*n* = 58)	Procedures (*n* = 78)	*p*-value
Revision timing			
Early (within 30 days)	33 (56.9%)	47 (60.3%)	0.012
Late (after 30 days)	25 (43.1%)	31 (39.7%)	0.089

*n*, number. Some patients underwent multiple revision procedures, resulting in more procedures (78) than patients (58). Mean revisions per patient: 1.34. Statistical methods: chi-square test for categorical comparisons. *p* < 0.05 considered statistically significant

The indications for revision and types of revision are summarized in Table 5. Early complications included stomal necrosis (36.2%, *n* = 21), retraction (32.7%, *n* = 19), and infection/abscess (24.1%, *n* = 14). Late complications included parastomal hernia (13.8%, *n* = 8) and stenosis (9.2%, *n* = 6). The most common procedure was complete revision (58.9%, *n* = 46), followed by local revision (29.5%, *n* = 23) and laparoscopic correction (11.5%, *n* = 9). Multiple revisions were performed in 34.5% of the patients: two revisions in 24.1% (*n* = 14) and three or more in 6.9% (*n* = 6). Stoma relocation varied by procedure type: local revisions maintained the original positioning, complete revisions relocated 85% of stomas, and laparoscopic corrections relocated all stomas to new sites.

**Table 5:** Revision procedures, indications, and types

Revision type	(*n* = 78)	Early revision indications		Late revision indications	
Complete revision	46 (58.9%)	Stoma necrosis	18 (23.1%)	Parastomal hernia	18 (10.3%)
Local revision	23 (29.5%)	Retraction	14 (24.3%)	Stenosis	6 (7.7%)
Laparoscopic correction	9 (11.5%)	Infection/abscess	11 (14.1%)	Infection/abscess vs	4 (5.1%)
Single revision	38 (65.5%)	Other early complicatons	4 (5.1%)	Prolapse	3 (3.8%)
Two revisions	14 (24.1%)				
Three or more revisions	6 (10.4%)				

### Clinical outcomes and risk factors

During index surgery, ICU hospitalization was higher in the revision group (82.8% vs. 61.4%), and postoperative hospital stay was significantly longer in the revision group (14.5 ± 6.2 vs. 10.2 ± 4.8 days; *p* = 0.005). Two-year mortality was significantly higher in the revision group (37.9% vs. 17.9%; *p* = 0.012). The primary cause of death was malignant disease progression in both groups (revision: 54.5%, *n* = 12; control: 58.5%, *n* = 31). However, sepsis-related deaths were notably higher in the revision group (22.7% vs. 11.3%), with 60% of deaths attributed to post-revision complications. Multivariable logistic regression identified independent risk factors for revision: transverse colostomy (OR: 2.12, 95% CI 1.18–3.81, *p* = 0.045), emergency surgery (OR: 1.96, 95% CI 1.09–3.52, *p* = 0.003), and preoperative albumin < 3.0 g/dl (OR: 2.34, 95% CI 1.25–4.38, *p* = 0.028). Age > 65 years, female sex, loop colostomy, and preoperative hemoglobin were significant in the univariate analysis but lost significance in the multivariable analysis (Table 6).

**Table 6:** Clinical outcomes and independent risk factors

Parameter	Revision group (*n* = 58)	Control group (*n* = 281)	Adjusted OR (95% CI)*	*p*-value
Demographic and clinical characteristics				
Age > 65 years, *n* (%)	35 (60.3)	142 (50.5)	1.68 (0.95–2.98)	0.021
Female sex, *n* (%)	31 (53.4)	111 (39.5)	1.52 (0.88–2.63)	0.045
Emergency surgery, *n* (%)	34 (58.6)	115 (40.9)	1.96 (1.09–3.52)	0.003
Preoperative albumin < 3.0 g/dl, *n* (%)	21 (34.5)	58 (14.7)	2.34 (1.25–4.38)	0.028
Preoperative hemoglobin < 11 g/dl, *n* (%)	24 (41.4)	78 (27.7)	1.58 (0.89–2.81)	0.035
Stoma characteristics				
Loop colostomy, *n* (%)	46 (79.3)	217 (77.2)	1.74 (0.87–3.47)	0.032
Transverse colostomy, *n* (%)	16 (27.5)	64 (23.8)	2.12 (1.18–3.81)	0.045
Treatment characteristics				
Neoadjuvant therapy, *n* (%)	13 (22.4)	48 (17.1)	–	0.242
Adjuvant therapy, *n* (%)	27 (46.6)	121 (43.1)	–	0.432
Clinical outcomes				
ICU admission, *n* (%)	48 (82.8)	180 (64.1)	2.45 (1.21–4.98)	0.001
Hospital stay (days, mean ± SD)	14.5 ± 6.2	10.2 ± 4.8	–	0.005
2-Year mortality, *n* (%)	22 (37.9)	53 (18.9)	1.89 (1.03–3.46)	0.012

*ICU*, intensive care unit; *OR*, odds ratio; *CI*, confidence interval; *SD*, standard deviation. Statistical analysis: Continuous variables were compared using Student’s *t*-test and categorical variables using the chi-square test. *Adjusted odds ratios were derived from multivariable logistic regression analysis using backward elimination. Variables with *p* ≤ 0.10 in univariate analysis were entered into the model

## Discussion

In this study, 58 patients who underwent colostomy revision were comprehensively examined, and their clinical characteristics and surgical outcomes were evaluated. The predominance of early revisions highlights the critical role of surgical techniques and perioperative optimization. This study presents a novel temporal analysis that distinguishes early ischemic complications from late mechanical complications and provides important data for developing preventive strategies.

In our study, 78 revision procedures were performed in 16.5% of colostomy patients, with an average of 1.3 interventions per patient. This rate is consistent with the literature [13, 14] but highlights the necessity of repeated revisions. The higher mean age in the revision group explains the increased risk, as age increases comorbidities and impairs healing [15, 16]. Similarly, Chaudhri et al. [17] found significantly higher complication rates in patients aged > 65 years.

Female sex was identified as an independent risk factor (53.4% vs. 39.5%, *p* = 0.045). This association can be explained by the higher subcutaneous adipose tissue thickness in the abdominal wall, which complicates stoma maturation, and gender-related differences in fascial healing capacity. Additionally, the effects of pelvic radiation on ostomy outcomes should be considered in female patients with a more frequent history of pelvic surgery and radiotherapy due to gynecological malignancies. Pelvic radiotherapy causes microvascular damage and fibrosis in the intestines, mesentery, and pelvic soft tissues, resulting in chronic hypoxia and impaired tissue healing [18]. This increases the risk of anastomotic leakage, stenosis, and obstruction, elevating the likelihood of stoma revision or inability to close [19]. Long-term studies have demonstrated significantly high persistent stoma rates in patients with rectal cancer receiving neoadjuvant radiation [20]. Furthermore, intense post-radiation fibrosis complicates intestinal mobilization and creates tension during ostomy placement. Current meta-analyses have confirmed that neoadjuvant chemoradiotherapy affects surgical outcomes and stoma requirements in locally advanced rectal cancer [21]. These mechanisms increase the susceptibility to stoma complications, including parastomal hernia, retraction, and skin problems, in patients with a history of pelvic radiotherapy. Preventive stoma planning requires a multidisciplinary approach during neoadjuvant treatment [22].

Emergency colostomies had a significantly higher revision risk (51.7% vs. 40.9%, *p* = 0.003), confirmed as an independent predictor (OR: 1.96, *p* = 0.025) [5, 14, 22]. Transverse colostomies also had a higher revision rate than sigmoid colostomies (24.7% vs. 13.8%, *p* = 0.045), consistent with the existing literature [23].

The embryological origin and vascular anatomy of the transverse colon explain its susceptibility. Located at the midgut-hindgut junction, its primary blood supply is provided by the middle colic artery. Variations in its branching or insufficiencies in the marginal arcade increase the risk of ischemia during stoma creation [24, 25]. The colon's larger diameter and thinner wall also elevate the risks of necrosis and retraction. The higher revision rate for loop colostomies is multifactorial; contributing factors include emergency surgery [26, 27], pressure necrosis from supporting rods, and inadequate fixation [28]. A larger fascial defect can also increase the incidence of parastomal hernias, prompting revision [29, 30]. Despite conflicting literature [27, 31], our study confirms a higher risk of revision with loop colostomies. Therefore, end colostomy may be preferred for patients requiring a permanent stoma.

One of the most distinctive findings of our study relates to the timing of revision procedures and the need for repeated interventions. The application of 78 total revision procedures in 58 patients revealed the complex nature of the complications in this population. In particular, the performance of 47 procedures in 33 patients during the early period suggests that early complications may have a more aggressive course and that initial interventions may be insufficient in some cases.

The distinct differences between the early and late complication models reflect different pathophysiological mechanisms. Early complications, such as necrosis and retraction, indicate acute ischemic or technical problems [31], whereas late complications, such as parastomal hernia and stenosis, are associated with chronic mechanical stress [27]. A notable finding was the high rate of multiple revisions; 34.5% of patients required more than one procedure, with an average of 1.34 revisions per patient. This indicates that initial revisions are often inadequate, particularly in the case of early complications. These findings emphasize the necessity of definitive rather than provisional initial surgical approaches, especially in high-risk patients. The cumulative burden of multiple interventions increases healthcare costs and contributes to patient morbidity and the observed high 2-year mortality rate.

Low preoperative albumin and hemoglobin levels indicated widespread malnutrition and chronic disease anemia in the revision group. Malnutrition impairs tissue healing and increases the risk of surgical site infection and anastomotic leakage [32]. Preoperative nutritional optimization reduces complications [33]. An albumin cutoff value < 3.0 g/dl has been established for surgical malnutrition according to the ESPEN guidelines [33, 34], and it has been determined that postoperative complications may increase significantly below this value. The implementation of preoperative optimization protocols recommended in the ESPEN guidelines is crucial [33]. However, hemoglobin level was not an independent risk factor in the multivariate analysis. The explanatory power of the model (Nagelkerke *R*^2^ = 0.347) indicates the presence of other unmeasured factors contributing to the risk of revision.

The high 2-year mortality rate in the revision group indicates that these patients have a fragile profile, both locally and systemically. More than half of the deaths in both groups were due to underlying malignant progression (revision, 54.5%; control, 58.5%). The most striking difference was mortality related to sepsis, which was 22.7% in the revision group and 11.3% in the control group. Importantly, 60% of sepsis deaths in the revision group were due to post-revision complications (anastomotic leakage, intra-abdominal abscess), whereas none of the sepsis deaths in the control group were stoma-related. This indicates that revision surgery poses a significant risk of morbidity and mortality [35]. These findings emphasize that patients requiring stoma revision need comprehensive medical evaluation and long-term follow-up.

Consistent with our study findings, a multifaceted approach is recommended to reduce the risk of colostomy revision. First, the identification of patients with advanced age, female sex, emergency surgery, malnutrition, and transverse colostomy indication as a high-risk group and the implementation of preoperative optimization protocols, including correction of nutritional status and aggressive treatment of anemia before elective surgery in these patients, are of critical importance. From a surgical technical perspective, attention should be paid to selecting a colon segment with an adequate vascular arcade supply in situations requiring transverse colostomy, with the sigmoid colon being the preferred choice when possible. If loop colostomy is to be performed, optimization of fascial defect size and consideration of prophylactic mesh application in patients at high risk of hernia are recommended [36, 37].

In the postoperative period, a more aggressive approach should be adopted to manage complications, such as necrosis and retraction, that develop within the first 30 days. Conservative treatment should be limited, early surgical revision should not be delayed, and the initial revision procedure should be as definitive as possible, considering the risk of repeated interventions. Particularly in complex cases, such as those involving patients with cancer, stoma care management by a multidisciplinary team consisting of surgeons, medical oncologists, stoma therapists, and dietitians plays a key role in preventing and managing complications. Finally, current surgical guidelines need to be strengthened with more specific recommendations regarding stoma creation in high-risk patient groups, prophylactic mesh use, and revision indications considering the findings of this study.

### Study limitations

This study had some limitations that should be considered. Owing to its retrospective design, there may be some shortcomings in data collection, and no causal relationships can be established. Its monocentric nature may limit the generalizability of our findings to other populations. Since the main purpose of our study was to create a profile of patients undergoing colostomy revision, complications that do not require revision (such as mild skin irritation, minor retraction, and asymptomatic hernia) were not systematically recorded. In addition, patients who were not subjected to surgery due to patient rejection or ongoing adjuvant treatment despite revision indications were not included in the study. Qualitative and technical details, such as the selection of stoma location, preoperative marking (by a stomatherapist nurse or surgeon), and whether the stoma was passed through or through the rectus muscle, were not consistently documented in the operation notes. Long-term QoL data were not included in this study. In addition, no prior formal sample size calculation was performed because of the retrospective nature of the study.

## Conclusion

Colostomy revision is a complex surgical challenge associated with high morbidity rates. The elevated mortality observed in patients undergoing revision surgery reflects their overall vulnerability and advanced underlying disease rather than revision surgery itself. Mortality analysis demonstrated that 54.5% of deaths resulted from malignancy progression, with only 13.6% directly attributable to revision surgery complications. Multivariable analysis identified preoperative hypoalbuminemia (< 3.0 g/dl), transverse colostomy location, and emergency surgery as independent risk factors. Early complications were predominantly ischemic (necrosis, retraction), whereas late complications were mainly mechanical (hernia, stenosis), necessitating distinct preventive approaches. A substantial proportion (34.5%) of patients required multiple revisions. Preoperative risk stratification, nutritional optimization, and preferential sigmoid over transverse colostomy placement in high-risk patients may reduce the revision burden. The systematic identification of high-risk patients and the implementation of optimal surgical techniques are essential.

## Data Availability

The datasets generated during and/or analyzed during the current study are available from the corresponding author upon reasonable request.
